# Supplementary material for: Towards an easier creation of three-dimensional data for embedding into scholarly 3D PDF (Portable Document Format) files
Source: PeerJ. 2015 Mar 3;3:e794. doi: 10.7717/peerj.794 (PMC4358654; doi:10.7717/peerj.794)
Supplement: Supplemental Information 1 — Binary files, module definition files and installation instructions. Using these files, the SaveU3D module and the MarkerListImport module can be added to an existing MeVisLab 2.6(.x) installation without the need to compile the source files. [file peerj-03-794-s001.zip › Visual Studio 2010/Documentation/Publish/ModuleReference/MarkerListImport.html]

MarkerListImport — MeVisLab documentation


### Navigation

# MarkerListImport¶

MLModule¶
:   |  |  |
    | --- | --- |
    | genre | Fields |
    | author | Axel Newe |
    | package | Community/General |
    | dll | MLBaseListExtensions |
    | definition | MLBaseListExtensions.def |
    | see also | XMarkerListContainer, ColoredMarkerListContainer |
    | keywords | ListBase, XMarker, ColoredMarker |

# Purpose¶

This module imports XMarkers from text files.

# Usage¶

Specify a file name, delimiter/separator characters and a filter (if applicable) and hit the "Load" button. The loaded XMarker list will then be provided at the output connector.

# Details¶

This module can be used to import XMarkers from text files, e.g. CSV files.

The coordinates must consist of 3 numbers (x, y, z coordinate) that are separated by a character that can be specified by means of the Number Delimiter field (or the Use space character as delimiter field, respectively). The decimal separator of each coordinate can be specified by means of the Decimal Separator field. Negative coordinate values must start with a minus sign ('-').

The XMarkers can be grouped by a preceding line that must be formatted like <Filter Tag> <Type #>, e.g.: 'PointSet 1'
The <Filter Tag> allows for filtering the input file. Only groups with the <Filter Tag> specified in the Filter field will be loaded.
The <Type #> allows for setting the type property of the XMarker. The <Type #> must be an integer and is applied to all XMarkers of the respective group.

Warning: if no filter is applied, only the <Type #> will be evaluated. Therefore, XMarkers in groups with different <Filter Tag> but the same <Type #> will get the same Type property if no filter is specified!

See the example network (ReadRawFile module) for an example input file with ';' as delimiter and ',' as decimal separator.

# Windows¶

## Default Panel¶

# Output Fields¶

## outMarkerList¶

name: outMarkerList, type: MLBase¶

# Parameter Fields¶

## Field Index¶

|  |
| --- |
| Auto Load: Bool |
| Decimal Separator: String |
| Filename: String |
| Filter: String |
| Load: Trigger |
| Number Delimiter: String |
| Unload: Trigger |
| Use space character as delimiter: Bool |

## Visible Fields¶

### Filename¶

name: filename, type: String¶

### Filter¶

name: filter, type: String¶

### Number Delimiter¶

name: numberDelimiter, type: String, default: ;¶

### Use space character as delimiter¶

name: numberDelimiterSpace, type: Bool, default: TRUE¶

### Decimal Separator¶

name: decimalSeparator, type: String, default: .¶

### Load¶

name: load, type: Trigger¶

### Unload¶

name: unload, type: Trigger¶

### Auto Load¶

name: autoLoad, type: Bool, default: FALSE¶

### Table Of Contents

- MarkerListImport
- Purpose
- Usage
- Details
- Windows
  - Default Panel
- Output Fields
  - outMarkerList
- Parameter Fields
  - Field Index
  - Visible Fields
    - Filename
    - Filter
    - Number Delimiter
    - Use space character as delimiter
    - Decimal Separator
    - Load
    - Unload
    - Auto Load

### Navigation

Created using Sphinx 1.0.4.
