# Supplementary material for: Towards an easier creation of three-dimensional data for embedding into scholarly 3D PDF (Portable Document Format) files
Source: PeerJ. 2015 Mar 3;3:e794. doi: 10.7717/peerj.794 (PMC4358654; doi:10.7717/peerj.794)
Supplement: Supplemental Information 1 — Binary files, module definition files and installation instructions. Using these files, the SaveU3D module and the MarkerListImport module can be added to an existing MeVisLab 2.6(.x) installation without the need to compile the source files. [file peerj-03-794-s001.zip › Visual Studio 2010/Documentation/Publish/ModuleReference/SaveU3D.html]

SaveU3D — MeVisLab documentation


### Navigation

# SaveU3D¶

MLModule¶
:   |  |  |
    | --- | --- |
    | genre | FileMain |
    | author | Axel Newe |
    | package | Community/General |
    | dll | MLPDF |
    | definition | MLPDF.def |
    | see also | WEMSaveAsU3D, ComposeWEMDescriptionForU3D |
    | keywords | PDF, U3D, WEM, XMarker, Fiber |

# Purpose¶

This module creates Universal 3D (U3D) files that can be imported into Portable Document Format (PDF) files for creating 3D annotations.

# Usage¶

Connect the inputs with the point set data (XMarkerList or ColoredMarkerList), the line set data (XMarkerList or ColoredMarkerList plus IndexPairList) and the mesh data (WEMs), specify the output (see "Details") and the file name and click the "Save" button.

# Details¶

The module creates U3D files as defined in Standard ECMA-363 (Universal 3D File Format), 4th edition (June 2007).

The following U3D elements can be added:

- PointSets
- LineSets
- Triangle meshes
- Meta data

The geometry data is fed in via the input connectors. However, since input data can be reused for multiple output models, the output models must be specified. This happens in the three tabs "Point Clouds", "Line Sets" and "Meshes". The general output geometry specification is the same for all three model types, but some details vary.

**Common Specification**

The general specification follows an XML-like principle. A new object specification is started with a single tag and the specification parameters are embraced with opening and closing tags.

The common tags are:

- *<ObjectName></ObjectName>*: This specifies the name under which the object is identified in the 3D model tree. Names do *not* need to be unique.
- *<GroupPath><GroupPath>*: This specifies the path where the object is placed in the 3D model tree. All necessary sub-branches are created automatically.
- *<Color><Color>*: This specifies the (diffuse) color of the object.
- *<SpecularColor><SpecularColor>*: This specifies the specular (reflective) color of the object.
- *<ModelVisibility><ModelVisibility>*: This specifies the model visibility (front visible, back visible, both sides visible or not visible).

These common tags are available for all object types. However, some object types do not evaluate all tags (e.g., a Point Cloud does not evaluate the <Color> tag). This is because the same specifications can also be used for other (non-U3D) export formats. In addition, future versions of the U3D standard *might* support the respective settings.

Colors are specified using triples (RGB) or quadrupels (RGBA) of floating point numbers bewteen 0 and 1.

**Point Cloud Specification**

A new point cloud is started by the *<PointSet>* tag. A closing tag is not necessary

The geometry data is taken from the inPointPositions input field, which accepts XMarkerLists or ColoredMarkerLists. Use the <PositionTypes> tag to filter which positions (Type ID numbers) of the input list shall be used. If no <PositionTypes> tag is specified, all positions are used. Multiple position types may be separated by commas.

**Line Set Specification**

A new line set is started by the *<LineSet>* tag. A closing tag is not necessary

The geometry data is taken from the inLinePositions input field (XMarkerList or ColoredMarkerList) and the inLineConnections input field (IndexPairList). Use the <PositionTypes> tag to filter which positions (Type ID numbers) of the input positions list shall be used. If no <PositionTypes> tag is specified, all positions are used. Use the <ConnectionTypes> tag to filter which connections/edges (Type ID numbers) of the input connections list shall be used. If no <ConnectionTypes> tag is specified, all connections are used. Instead of specifying Type ID numbers, the <ConnectionTypes> tag may also contain the *simple* keyword. In this case, the connections are generated automatically by simply connecting each position with the next in the list. Multiple position types and connection types may be separated by commas.

**Mesh Specification**

A new mesh is started by the *<Mesh>* tag. A closing tag is not necessary

The geometry data is taken from the inWEM input field, which accepts a WEM. Use the <WEMLabel> tag to filter which WEM (selected by its Label) of the input WEM shall be used.

**Meta Data**

U3D allows for inclusion of meta data. Since meta data is no geometry data, it is specified purely via the UI.

A new meta data entry is started by the *<MetaData>* tag. A closing tag is not necessary.

Each meta data entry needs a key and a value. These are specified using the

- *<MetaDataKey></MetaDataKey>* and
- *<MetaDataValue></MetaDataValue>*

tags.

# Tips¶

There is a simple mode available, which creates default 3D models from the input data. If you only connect input geometry data (e.g. a WEM or a positions list) and activate the respective "Enable Simple Mode" checkbox, all geometry data is automatically converted into 3D objects using default settings. In case of WEMs, the WEM description is also parsed. You can use the ComposeWEMDescriptionForU3D module to create suitable WEM descriptions.

You can use a FiberSetToXMarker module to connect a fiber set to the inLinePositions input.

Have a look at the example network which demonstrates all features.

This module requires the MLBaseListExtensions.

# Windows¶

## Main Panel¶

The main panel contains a "Default Settings" tab. This tab contains the default object specifications that pitch in if they are not explicitly specified in the respective object type tabs.

## Specification Generator Panel¶

For your convenience, the module has a built-in specification generator. You can use it to specify 3D object settings using standard MeVisLab fields. Use the "Add" button to add the currently displayed specification to the respective specifications in the main module.

# Input Fields¶

## inWEM¶

name: inWEM, type: MLBase¶
:   Contains the mesh geometry data.
    Allowed: WEMs.

## inPointPositions¶

name: inPointPositions, type: MLBase¶
:   Contains the point cloud positions geometry data.
    Allowed: XMarkerList and ColoredMarkerList.

## inLinePositions¶

name: inLinePositions, type: MLBase¶
:   Contains the line set positions (nodes) geometry data.
    Allowed: XMarkerList and ColoredMarkerList.

## inLineConnections¶

name: inLineConnections, type: MLBase¶
:   Contains the line set connections (edges) geometry data.
    Allowed: IndexPairList.

# Parameter Fields¶

## Field Index¶

|  |  |  |
| --- | --- | --- |
| Add: Trigger | Enble Simple Mode (add all patches from input wem and parse WEM description for details).: Bool | Object Group Path: String |
| Add default bounding box meta data: Bool | Enble Simple Mode (collect all positions from input field and assemble one line set with default settings).: Bool | Object Name: String |
| Add default Light Node: Bool | Enble Simple Mode (collect all positions from input field and assemble one point cloud with default settings).: Bool | Object Type: Enum |
| Add default View Node: Bool | Filename: String | Point Cloud Specification: String |
| apply: Trigger | isProcessing: Bool | Position Types: String |
| Auto Apply: Bool | lightsSpecification: String | Prefix for Line Sets Without Name: String |
| Auto Update: Bool | Line Set Specification: String | Prefix for Meshes Without Name: String |
| autoClear: Bool | listenToFinishingNotifications: Bool | Prefix for Point Clouds Without Name: String |
| Connection Types: String | listenToRepaintNotifications: Bool | progress: Float |
| Default Light Color: Color | listenToSelectionChangedNotifications: Bool | Save: Trigger |
| Default Light Intensity: Double | Mesh Specification: String | selectedTab: Integer |
| Default Light Name: String | Meta Data Key: String | Specular Color: Color |
| Default Material Alpha: Float | Meta Data Specification: String | Status: String |
| Default Material Ambient Color: Color | Meta Data Value: String | Use Default Color: Bool |
| Default Material Color: Color | Model Visibility: Enum | Use Default Specular Color: Bool |
| Default Material Emissive Color: Color | New Specification: String | viewsSpecification: String |
| Default Material Specular Color: Color | newSpecificationOutputValid: Bool | WEM Label: String |
| Default View Name: String | Object Alpha: Float |  |
| elapsedTime: Float | Object Color: Color |  |

## Visible Fields¶

### Auto Apply¶

name: autoApply, type: Bool, default: FALSE¶

### Auto Update¶

name: autoUpdate, type: Bool, default: FALSE¶

### Enble Simple Mode (collect all positions from input field and assemble one point cloud with default settings).¶

name: simpleModePointSet, type: Bool, default: FALSE¶

### Enble Simple Mode (collect all positions from input field and assemble one line set with default settings).¶

name: simpleModeLineSet, type: Bool, default: FALSE¶

### Enble Simple Mode (add all patches from input wem and parse WEM description for details).¶

name: simpleModeMesh, type: Bool, default: FALSE¶

### Prefix for Point Clouds Without Name¶

name: modelPrefixPointClouds, type: String, default: PointSet¶

### Prefix for Line Sets Without Name¶

name: modelPrefixLineSets, type: String, default: LineSet¶

### Prefix for Meshes Without Name¶

name: modelPrefixMeshes, type: String, default: Mesh¶

### Point Cloud Specification¶

name: pointCloudSpecification, type: String¶

### Line Set Specification¶

name: lineSetSpecification, type: String¶

### Mesh Specification¶

name: meshSpecification, type: String¶

### Meta Data Specification¶

name: metaDataSpecification, type: String¶

### Default View Name¶

name: defaultViewName, type: String, default: DefaultView¶

### Default Light Name¶

name: defaultLightName, type: String, default: DefaultAmbientLight¶

### Default Material Color¶

name: defaultMaterialDiffuseColor, type: Color, default: 0.649999976158142 0.649999976158142 0.649999976158142¶

### Default Material Specular Color¶

name: defaultMaterialSpecularColor, type: Color, default: 0.75 0.75 0.75¶

### Default Material Ambient Color¶

name: defaultMaterialAmbientColor, type: Color, default: 0 0 0¶

### Default Material Emissive Color¶

name: defaultMaterialEmissiveColor, type: Color, default: 0 0 0¶

### Default Material Alpha¶

name: defaultMaterialAlpha, type: Float, default: 1, minimum: 0, maximum: 1¶

### Default Light Color¶

name: defaultLightColor, type: Color, default: 1 1 1¶

### Default Light Intensity¶

name: defaultLightIntensity, type: Double, default: 1, minimum: 0, maximum: 1¶

### Add default bounding box meta data¶

name: defaultBoundingBoxMetaData, type: Bool, default: TRUE¶

### Add default View Node¶

name: addDefaultViewNode, type: Bool, default: TRUE¶

### Add default Light Node¶

name: addDefaultLightNode, type: Bool, default: TRUE¶

### Filename¶

name: filename, type: String¶

### Save¶

name: save, type: Trigger¶

### Status¶

name: status, type: String, default: Idle.¶

### Object Type¶

name: newSpecificationType, type: Enum, default: SPECTYPE\_POINTCLOUD¶

Values:

| Title | Name |
| --- | --- |
| Point Cloud | SPECTYPE\_POINTCLOUD |
| Line Set | SPECTYPE\_LINESET |
| Mesh | SPECTYPE\_MESH |
| Meta Data | SPECTYPE\_METADATA |

### Object Name¶

name: newSpecificationObjectName, type: String¶

### Object Group Path¶

name: newSpecificationGroupPath, type: String¶

### Use Default Color¶

name: newSpecificationUseDefaultColor, type: Bool, default: TRUE¶

### Use Default Specular Color¶

name: newSpecificationUseDefaultSpecularColor, type: Bool, default: TRUE¶

### Object Color¶

name: newSpecificationColor, type: Color, default: 0.651 0.651 0.651¶

### Object Alpha¶

name: newSpecificationColorAlpha, type: Float, default: 1, minimum: 0, maximum: 1¶

### Specular Color¶

name: newSpecificationSpecularColor, type: Color, default: 0.75 0.75 0.75¶

### Model Visibility¶

name: newSpecificationModelVisibility, type: Enum, default: SPECTYPE\_FRONTANDBACKVISIBLE¶

Values:

| Title | Name |
| --- | --- |
| Not Visible | VISIBILITY\_NOTVISIBLE |
| Front Visible | SPECTYPE\_FRONTVISIBLE |
| Back Visible | SPECTYPE\_BACKVISIBLE |
| Front & Back Visible | SPECTYPE\_FRONTANDBACKVISIBLE |

### WEM Label¶

name: newSpecificationWEMLabel, type: String¶

### Position Types¶

name: newSpecificationPositionTypes, type: String¶

### Connection Types¶

name: newSpecificationConnectionTypes, type: String¶

### Meta Data Key¶

name: newSpecificationMetaDataKey, type: String¶

### Meta Data Value¶

name: newSpecificationMetaDataValue, type: String¶

### New Specification¶

name: newSpecification, type: String, default: <PointSet>, <ModelVisibility>3</ModelVisibility>, ,¶

### Add¶

name: newSpecificationAdd, type: Trigger¶

## Hidden Fields¶

### isProcessing¶

name: isProcessing, type: Bool, default: FALSE¶

### elapsedTime¶

name: elapsedTime, type: Float, default: 0¶

### autoClear¶

name: autoClear, type: Bool, default: TRUE¶

### apply¶

name: apply, type: Trigger¶

### listenToFinishingNotifications¶

name: listenToFinishingNotifications, type: Bool, default: FALSE¶

### listenToRepaintNotifications¶

name: listenToRepaintNotifications, type: Bool, default: FALSE¶

### listenToSelectionChangedNotifications¶

name: listenToSelectionChangedNotifications, type: Bool, default: FALSE¶

### viewsSpecification¶

name: viewsSpecification, type: String¶

### lightsSpecification¶

name: lightsSpecification, type: String¶

### progress¶

name: progress, type: Float, persistent: no¶

### newSpecificationOutputValid¶

name: newSpecificationOutputValid, type: Bool, default: TRUE¶

### selectedTab¶

name: selectedTab, type: Integer, default: 0, minimum: 0, maximum: 3¶

### Table Of Contents

- SaveU3D
- Purpose
- Usage
- Details
- Tips
- Windows
  - Main Panel
  - Specification Generator Panel
- Input Fields
  - inWEM
  - inPointPositions
  - inLinePositions
  - inLineConnections
- Parameter Fields
  - Field Index
  - Visible Fields
    - Auto Apply
    - Auto Update
    - Enble Simple Mode (collect all positions from input field and assemble one point cloud with default settings).
    - Enble Simple Mode (collect all positions from input field and assemble one line set with default settings).
    - Enble Simple Mode (add all patches from input wem and parse WEM description for details).
    - Prefix for Point Clouds Without Name
    - Prefix for Line Sets Without Name
    - Prefix for Meshes Without Name
    - Point Cloud Specification
    - Line Set Specification
    - Mesh Specification
    - Meta Data Specification
    - Default View Name
    - Default Light Name
    - Default Material Color
    - Default Material Specular Color
    - Default Material Ambient Color
    - Default Material Emissive Color
    - Default Material Alpha
    - Default Light Color
    - Default Light Intensity
    - Add default bounding box meta data
    - Add default View Node
    - Add default Light Node
    - Filename
    - Save
    - Status
    - Object Type
    - Object Name
    - Object Group Path
    - Use Default Color
    - Use Default Specular Color
    - Object Color
    - Object Alpha
    - Specular Color
    - Model Visibility
    - WEM Label
    - Position Types
    - Connection Types
    - Meta Data Key
    - Meta Data Value
    - New Specification
    - Add
  - Hidden Fields
    - isProcessing
    - elapsedTime
    - autoClear
    - apply
    - listenToFinishingNotifications
    - listenToRepaintNotifications
    - listenToSelectionChangedNotifications
    - viewsSpecification
    - lightsSpecification
    - progress
    - newSpecificationOutputValid
    - selectedTab

### Navigation

Created using Sphinx 1.0.4.
