# Supplementary figures and images for: Towards an easier creation of three-dimensional data for embedding into scholarly 3D PDF (Portable Document Format) files
Source: PeerJ. 2015 Mar 3;3:e794. doi: 10.7717/peerj.794 (PMC4358654; doi:10.7717/peerj.794)

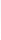

Supplement: Supplemental Information 1 — Binary files, module definition files and installation instructions. Using these files, the SaveU3D module and the MarkerListImport module can be added to an existing MeVisLab 2.6(.x) installation without the need to compile the source files. [file peerj-03-794-s001.zip › Visual Studio 2010/Documentation/Publish/ModuleReference/_static/contents.png]

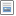

Supplement: Supplemental Information 1 — Binary files, module definition files and installation instructions. Using these files, the SaveU3D module and the MarkerListImport module can be added to an existing MeVisLab 2.6(.x) installation without the need to compile the source files. [file peerj-03-794-s001.zip › Visual Studio 2010/Documentation/Publish/ModuleReference/_static/file.png]

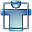

Supplement: Supplemental Information 1 — Binary files, module definition files and installation instructions. Using these files, the SaveU3D module and the MarkerListImport module can be added to an existing MeVisLab 2.6(.x) installation without the need to compile the source files. [file peerj-03-794-s001.zip › Visual Studio 2010/Documentation/Publish/ModuleReference/_static/MeVisLab.png]

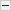

Supplement: Supplemental Information 1 — Binary files, module definition files and installation instructions. Using these files, the SaveU3D module and the MarkerListImport module can be added to an existing MeVisLab 2.6(.x) installation without the need to compile the source files. [file peerj-03-794-s001.zip › Visual Studio 2010/Documentation/Publish/ModuleReference/_static/minus.png]

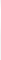

Supplement: Supplemental Information 1 — Binary files, module definition files and installation instructions. Using these files, the SaveU3D module and the MarkerListImport module can be added to an existing MeVisLab 2.6(.x) installation without the need to compile the source files. [file peerj-03-794-s001.zip › Visual Studio 2010/Documentation/Publish/ModuleReference/_static/navigation.png]

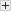

Supplement: Supplemental Information 1 — Binary files, module definition files and installation instructions. Using these files, the SaveU3D module and the MarkerListImport module can be added to an existing MeVisLab 2.6(.x) installation without the need to compile the source files. [file peerj-03-794-s001.zip › Visual Studio 2010/Documentation/Publish/ModuleReference/_static/plus.png]

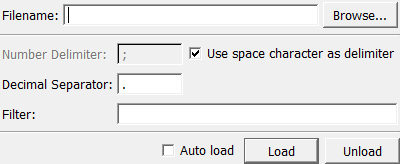

Supplement: Supplemental Information 1 — Binary files, module definition files and installation instructions. Using these files, the SaveU3D module and the MarkerListImport module can be added to an existing MeVisLab 2.6(.x) installation without the need to compile the source files. [file peerj-03-794-s001.zip › Visual Studio 2010/Modules/ML/MLBaseListExtensions/mhelp/Images/Screenshots/MarkerListImport._default.png]

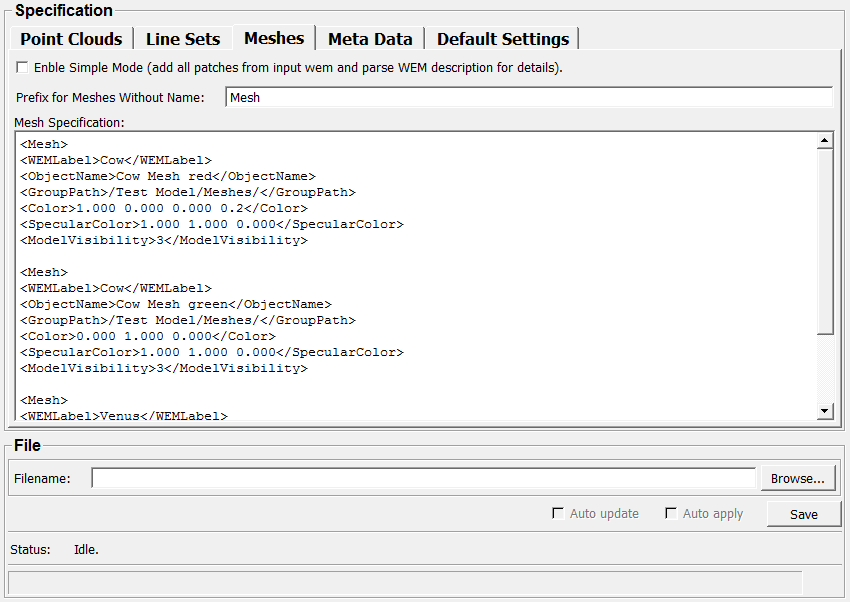

Supplement: Supplemental Information 1 — Binary files, module definition files and installation instructions. Using these files, the SaveU3D module and the MarkerListImport module can be added to an existing MeVisLab 2.6(.x) installation without the need to compile the source files. [file peerj-03-794-s001.zip › Visual Studio 2010/Modules/ML/MLPDF/mhelp/Images/Screenshots/SaveU3D.Main-Panel.png]

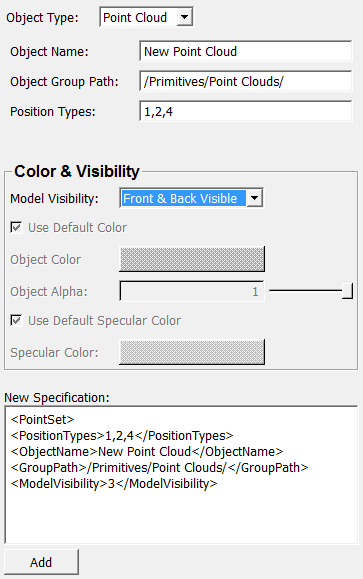

Supplement: Supplemental Information 1 — Binary files, module definition files and installation instructions. Using these files, the SaveU3D module and the MarkerListImport module can be added to an existing MeVisLab 2.6(.x) installation without the need to compile the source files. [file peerj-03-794-s001.zip › Visual Studio 2010/Modules/ML/MLPDF/mhelp/Images/Screenshots/SaveU3D.Specification-GeneratorPanel.png]
