# Supplementary material for: Towards an easier creation of three-dimensional data for embedding into scholarly 3D PDF (Portable Document Format) files
Source: PeerJ. 2015 Mar 3;3:e794. doi: 10.7717/peerj.794 (PMC4358654; doi:10.7717/peerj.794)

Towards an easier creation of three-dimensional Data for embedding into scholarly 3D PDF (Portable Document Format) files

Axel Newe

Supplementary File S2

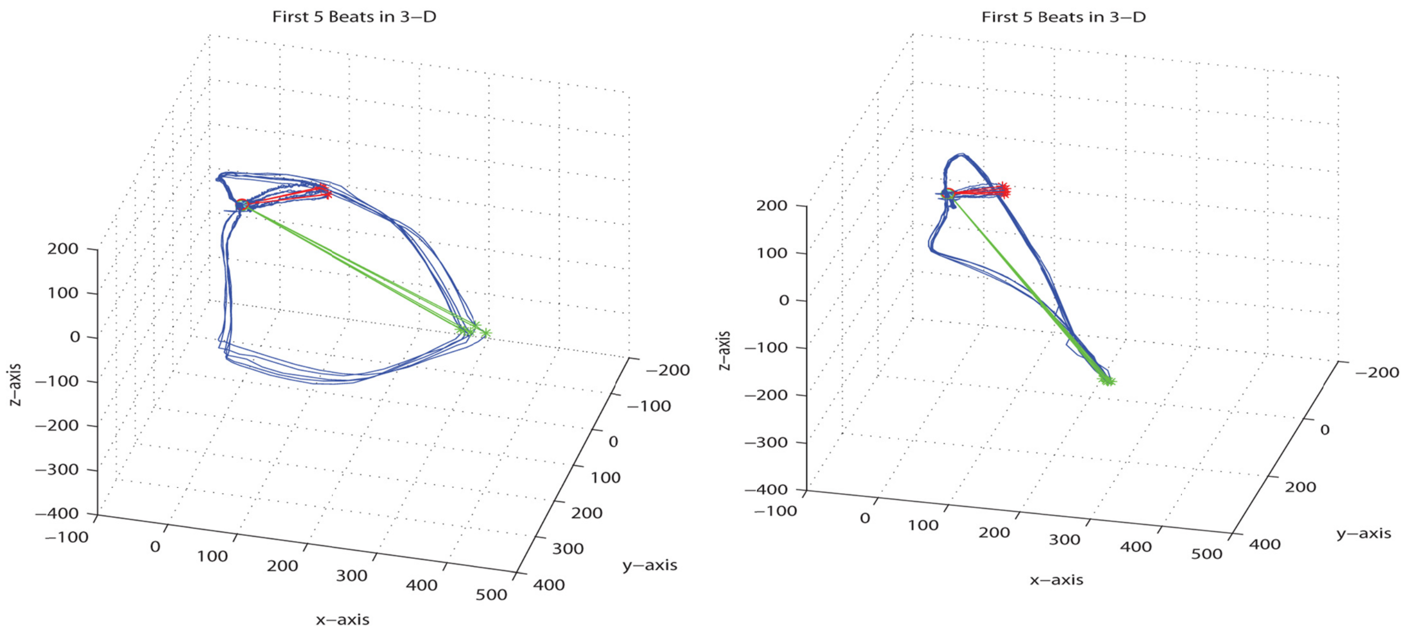

The above VCG illustrations were published in (Sur, Han & Tereshchenko, 2013). The original figure image is licensed under CC-BY 4.0 (http://creativecommons.org/licenses/by/4.0/).

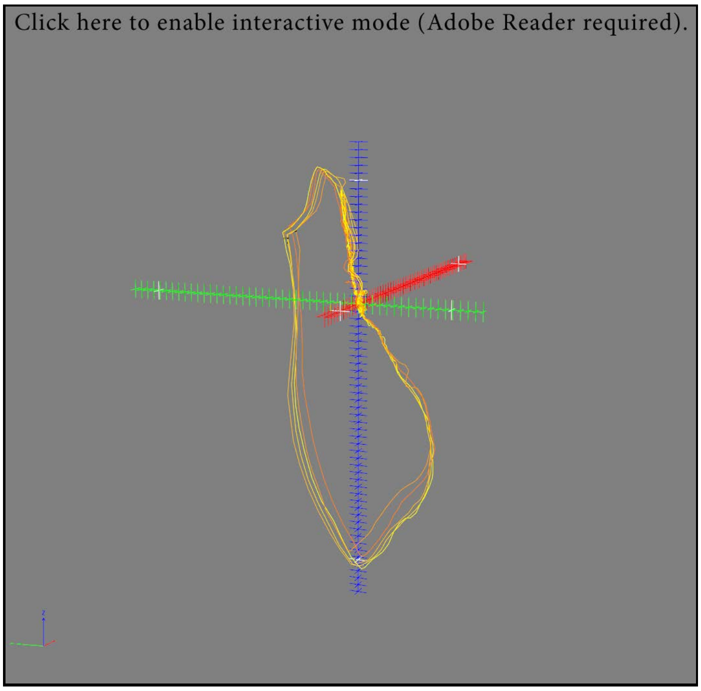

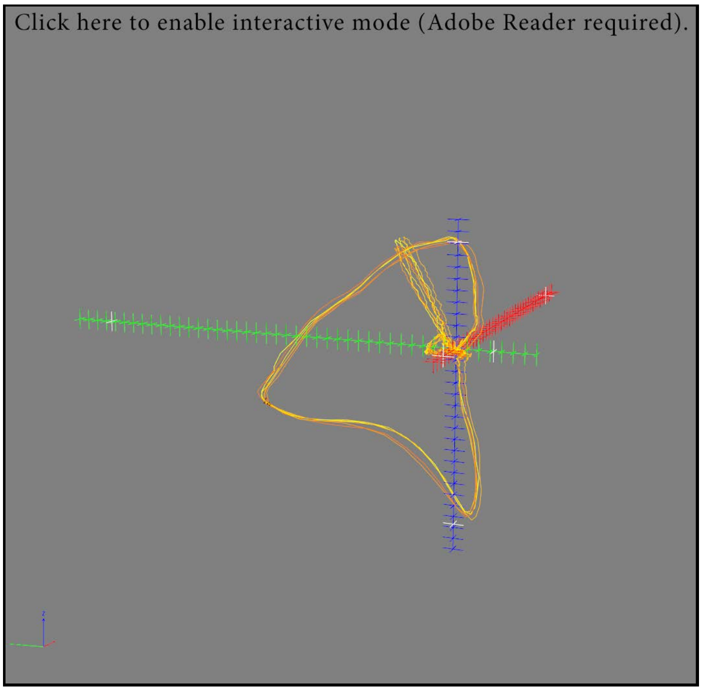

Supplement: Supplemental Information 2 — Two example U3D files of the vectorcardiograms shown in Figure 4 and the resulting 3D PDF. [file peerj-03-794-s002.zip › VCG-Examples.3d.pdf]
