# Supplementary material for: Towards an easier creation of three-dimensional data for embedding into scholarly 3D PDF (Portable Document Format) files
Source: PeerJ. 2015 Mar 3;3:e794. doi: 10.7717/peerj.794 (PMC4358654; doi:10.7717/peerj.794)
Supplement: Supplemental Information 3 — An example U3D file of lumbar vertebrae as shown in Fig. 5 and the resulting 3D PDF. [file peerj-03-794-s003.zip › Vertebrae-Example.3d.pdf]

Towards an easier creation of three-dimensional Data for embedding into scholarly 3D PDF (Portable Document Format) files

Axel Newe

Supplementary File S3

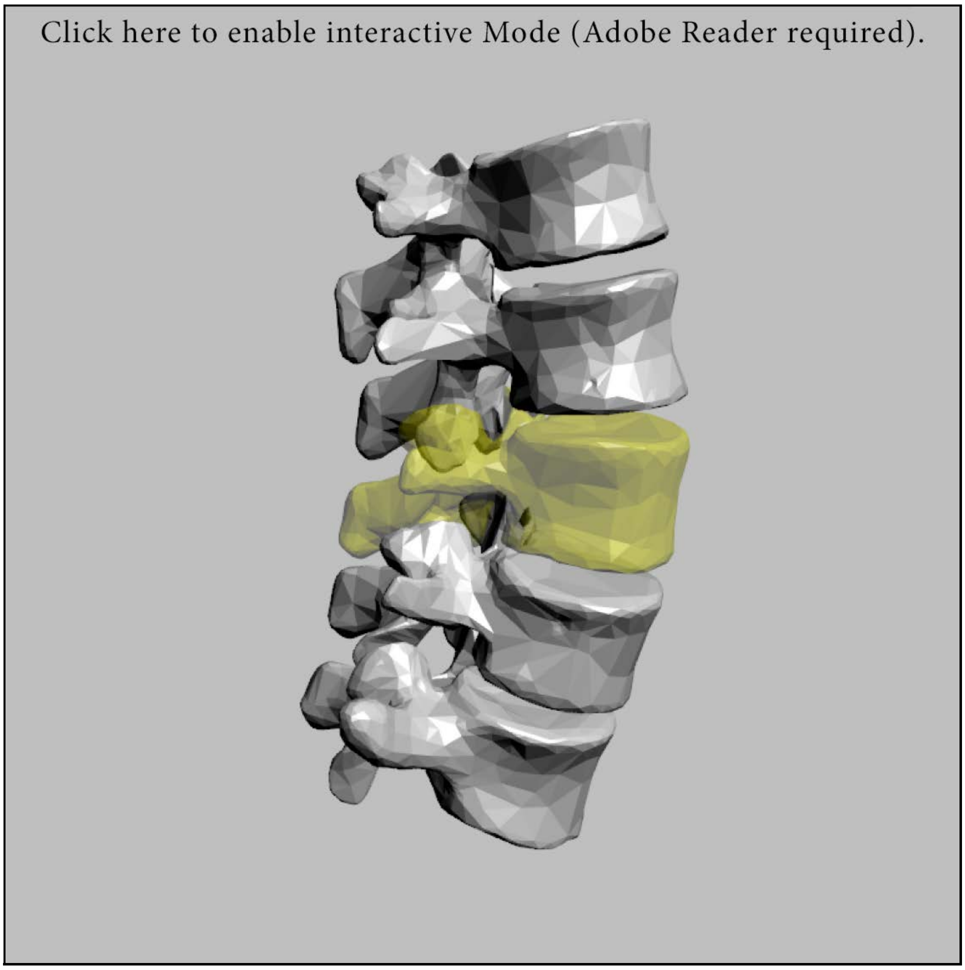

Example of 3D surface meshes converted by MeVisLab using the SaveU3D module.
One mesh has been exported using color and transparency. The original mesh data was downloaded in Wavefront OBJ file format from BodyParts3D, The Database Center for Life Science (Mitsuhashi et al., 2009) and is licensed under CC-BY-SA 2.1 Japan.
